# The Period When Teeth Begin to Form

**Published:** 1872-08

**Authors:** Geo. B. Harriman

**Affiliations:** Boston, Mass.


### Dental Register.

VOL. XXVI.] AUGUST, 1872. No. 8.]

### COMMUNICATIONS.

### The Period When Teeth Begin to Form.

PROF. GEO. B. HARRIMAN, BOSTON, MASS.

In closing my last article an allusion was made to positions in dentistry, which are held by both professors and authors from which I dissent. I have come to present conclusions after long and close investigation, and study, and am satisfied they rest on a basis which corresponding toil and effort on the part of others will sustain as both sound and truthful.

In the present article it is my purpose to consider the *matter* of *dental development* or the period at which the formation of the teeth begins.

In relation to this matter there are various theories. Writers of acknowledge ability and erudition both in general and anatomical science advocate different hypotheses. The position more commonly received and taken is that of Dr. Goodsir, who is regarded as indisputable authority. He maintained "that in the sixth week of fœtal life a narrow groove is found in the upper jaw of the human embryo, between the lips and the rudimentary palate, which is speedily divided into two by a ridge that subsequently becomes the external alveolar process, and it is in the inner groove where the germs of the teeth afterwards appear. This is the primitive dental groove. About the seventh week an avoidal papilla composed of a granular substance appears on the floor of the grove near the posterior terminus, which is the germ of the anterior superior milk molar tooth." In this manner he delineates the process of growth from week to week. I have looked very carefully into this matter, throughly examined the fœtus of almost every mouth and have come to the conclusion that but very little, if any, accurate information of the teeth can be derived at so early a period of fœtal existence. In an especial manner is there great uncertainty relative to this point among the people of our country. The character of our food and the methods of cooking, especially of bread, is very unfavorable to the formation of bone and tooth substance, and thus a barrier at once exists to the development of sound and durable teeth.

Without further remark on this matter for the present, I would observe that from repeated examinations of the fœtus in weekly stages, I have been unable to detect any groove where the germs of the teeth appear as advocated by Dr. Goodsir and others. Nor have my repeated examinations enabled me to discover in the tenth or twelfth week, signs or indications of the structure of any part of the tooth. The earliest period of any deposit of lime salts in the *cartilege* of the jaw, which I have been able to discern, is from the tenth or the twelfth week, and I have not been able to trace any indication whatever of the formation of the teeth prior to the seventh month, and then I could not discern anything like the substance of a milk molar tooth although Dr. Goodsir alleges the papilla of the first molar is perceptible at the seventh week of fœtal life. If a section is made through the center of the superior or inferior maxillary at about the seventh month, we find that the central incisors are beginning to form, and at the eighth month there is such a deposit of calcarous salts as to be seen with the eye.

In my investigations I have examined more than one hundred and forty embyros from four *weeks to fully developed fœtal life*, and I am stating facts as they presented themselves to me through the microscope, made by R. B. Tolles, of Boston, and which magnifies from one hundred to ten thousand diameter. The one-fiftieth immersion objective that I own made by him has been compared by Dr. Woodward, of Washington,

D. C., with the best one-fiftieth of Powell and Leland, of London, and is pronounced as far superior to anything yet produced of so high magnifying capacity. By reason of my having been so thorough and searching in my investigations, I feel very positive in my positions and am certain such phenomena as Dr. Goodsir describes could not have escaped my notice did such exist.

More than this if we look at the cuts representing the development and formation of the teeth as presented in the books, and compare these with the real formations, the most ignorant in the dental profession could not fail to detect the untruthfulness of the drawings. The cuts I present in these articles I intend to have as faithful to nature as I can possibly make them. A truthful drawning of what a microscopist has seen recently, may be valuable when compared with other investigators in years hence, whose drawnings perhaps may be more perfect as science advances, and errors be more readily detected. By description alone writers may so express themselves as to render it exceedingly doubtful what their opinions are, but a truthful and accurate drawing can not be overestimated.

I am not to be understood as affirming that there are no exceptional cases to what I have stated in regard to the time when the teeth commence to form.

There are several cases recorded where children have been born with erupted teeth. I have knowledge of two families where in one case a boy had two teeth at his birth and another had one; but these are exceptional cases, as much so as the birth of children with one arm, or with one leg shorter than the other, or with six fingers on one hand, or with a hair lip. It is a well authenticated fact that as a general thing there is no eruption of teeth in children before the fourth or sixth month of life. However interesting to the physiologist may be the inquiry for the period at which the formation of the teeth commences, it is of far less moment than the question what makes healthy, strong, and *altogether appropriate for their great end, the parts in which this formaton begins, continues and perfects.* Here is a most interesting and important department in the labors of the dentist. Say not here is the province of the physiologist, of the physician, and not of the dentist. I repeat it is one of the duties of the dentist in these days of light and knowledge to be thoroughly acquainted with everything directly or indirectly appertaining to the teeth. He should *especially* know all the elements of strength and power in the *bed* where the teeth are lodged. If he has this knowledge he will also know what will give strength, duration, and beauty to the teeth themselves when they are fully developed and formed. To gain this knowledge continued study and investigation will be needful.

Individuals indisposed to this latter duty had better retire from the profession.

